# Modulation of acoustomechanical instability and bifurcation behavior of soft materials

**DOI:** 10.1038/s41598-018-34971-x

**Published:** 2018-11-09

**Authors:** Fengxian Xin, Tian Jian Lu

**Affiliations:** 1 State Key Laboratory for Strength and Vibration of Mechanical Structures, Xi’an Jiaotong University, Xi’an, 710049 P. R. China; 2 MOE Key Laboratory for Multifunctional Materials and Structures, Xi’an Jiaotong University, Xi’an, 710049 P. R. China; 3 State Key Laboratory of Mechanics and Control of Mechanical Structures, Nanjing University of Aeronautics and Astronautics, Nanjing, 210016 P. R. China

## Abstract

We demonstrate acoustically triggered giant deformation of soft materials, and reveal the snap-through instability and bifurcation behavior of soft materials in nonlinear deformation regime in response to combined loading of mechanical and acoustic radiation forces. Our theoretical results suggest that acoustomechanical instability and bifurcation can be readily modulated by varying either the mechanical or acoustic force. This modulation functionality arises from the sensitivity of acoustic wave propagation to nonlinear deformation of soft material, particularly to ratio of initial geometrical size of soft material to acoustic wavelength in the material. The tunable acoustomechanical instability and bifurcation behavior of soft materials enables innovative design of programmable mechanical metamaterials. PACS numbers: 43.35.+d, 43.25.+y, 46.70.De, 61.41.+e.

## Introduction

Nonlinear deformation of soft materials in response to various external stimuli is often accompanied with elastic stability and bifurcation phenomena, which are traditionally averted for they represent mechanical failure. Recently, however, there is arising interest in harnessing such elastic instability and bifurcation to enable new functionalities of soft materials, such as triggering giant deformation^1,2^, auxetic material design^3^, amplifying response^4^, tunable mechanical response^5^, and acoustic rectification^6^. Particular focus has been placed upon developing soft materials with programmable behaviors, which often show non-monotonic and discontinuous responses or instabilities^5,7^. Nonetheless, a remarkable limitation to realize these programmable behaviors is that architected microstructures with beam elements are typically required.

We address this deficiency by demonstrating a novel strategy for tunable instability and bifurcation of homogeneous and isotropic soft materials by manipulating the acoustic radiation forces^8–11^. The acoustic radiation force is interpreted as a time-averaged steady force, which is generated when high-frequency acoustic wave such as ultrasound propagates in a nonlinear medium^12–14^. Harnessing the sensitivity of acoustic radiation force to material configuration, we demonstrate first the occurrence of acoustomechanical instability and bifurcation of soft material when it is subjected to combined mechanical and acoustical loads, and then realize their modulation via programmable mechanical force and acoustic input, respectively. The fast and non-contact modulation of acoustic input enables instantaneous and highly controllable nonlinear behavior of soft materials, with promising applications in medical devices, microfluidic actuators, adaptive robots, and so like.

## Theoretical Model

The propagation of ultrasonic wave from surrounding medium into a soft material generates acoustic radiation force due to acoustic momentum transfer inside the medium and material as well as at the interface between the two, which manifests in the form of a second-rank stress tensor if we consider a micro-cubic element of the material. This acoustic radiation stress tensor can be expressed as^15–20^

1 $$\langle {T}_{ij}\rangle =[\frac{\langle {p}^{2}\rangle }{2{\rho }_{a}{c}_{a}^{2}}-\frac{{\rho }_{a}\langle {u}_{k}\cdot {u}_{k}\rangle }{2}]\,{\delta }_{ij}+{\rho }_{a}\langle {u}_{i}\cdot {u}_{j}\rangle $$

where *P* is acoustic pressure, *ρ*_*a*_ medium density, *c*_*a*_ acoustic speed in medium, *u*_*i*_, *u*_*j*_ and *u*_*k*_ velocity components, and 〈⋅〉 denotes time-average manipulation over an oscillation cycle. Therefore, the acoustical radiation stress is scaled as ${p}_{0}^{2}/({\rho }_{a}{c}_{a}^{2})$, *p*_0_ being amplitude of input sound pressure. Commonly, as a focused acoustic pressure lies between 0.1 and 4 MPa, the corresponding acoustic radiation stress ranges from 70247 Pa to 112 MPa in air, and from 4.44 Pa to 7111 Pa in water. If one note that the elastic modulus of soft materials is generally several times kPa, the acoustic radiation stress is lager enough to cause material deformation: even large nonlinear deformation is possible^13,21–24^.

**Figure 1:**
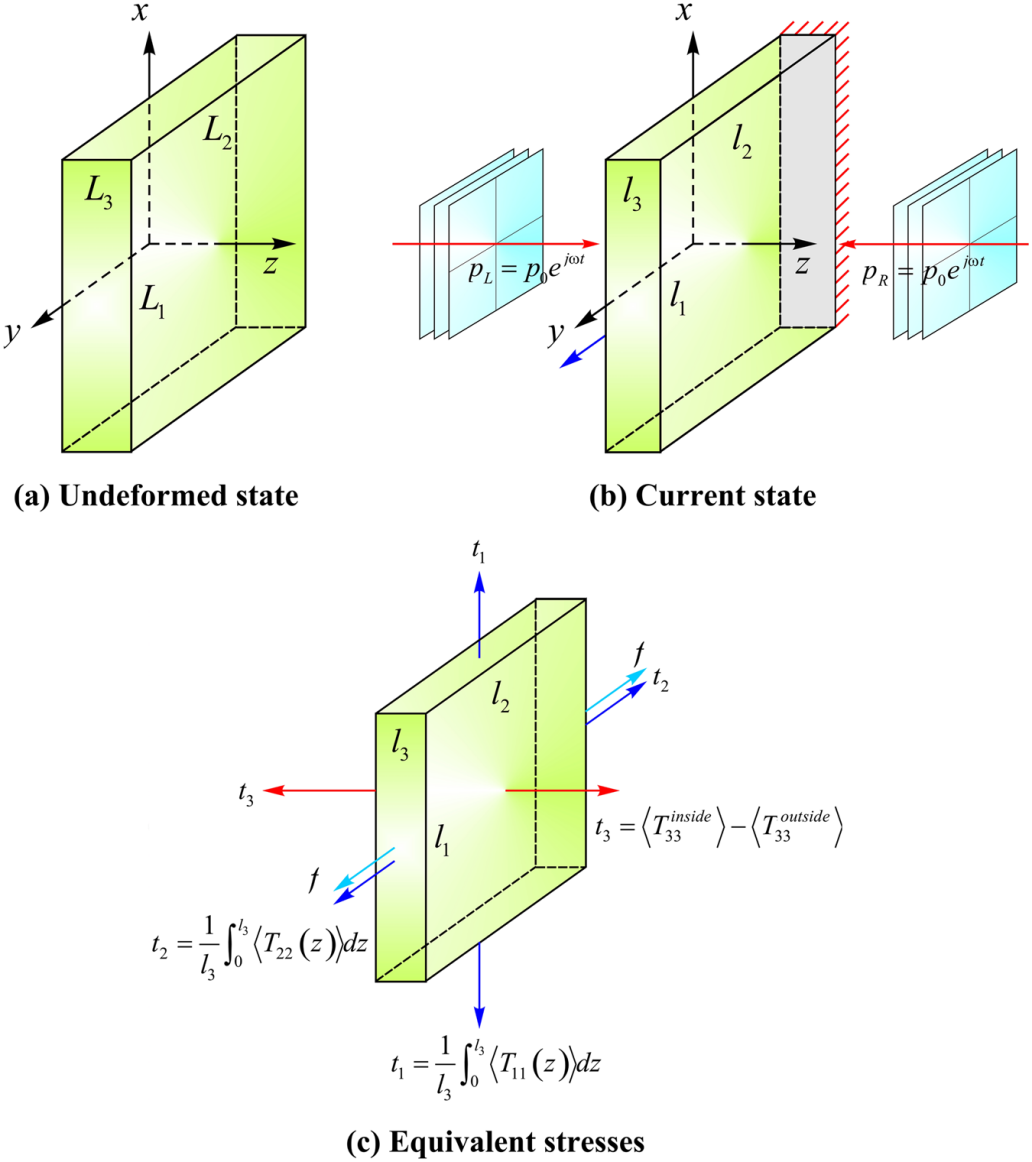
Deformation of soft material sheet by acoustical radiation forces: (**a**) undeformed sheet of dimensions (*L*_1_, *L*_2_, *L*_3_); (**b**) the sheet deforms to dimensions (*l*_1_, *l*_2_, *l*_3_) under two opposing sound pressure fields *p*_*L*_ = *p*_*L*0_*e*^*jωt*^ and *p*_*R*_ = *p*_*R*0_*e*^*jωt*^; (**c**) equivalent stresses induced by acoustic radiation forces.

To be more specific, we consider in Fig. 1a thin sheet of soft material. Let its outside and inside media have acoustic impedance *ρ*_1_*c*_1_ and *ρ*_2_*c*_2_, respectively. The thickness of the sheet is considered comparable to the wavelength of acoustic wave propagating in the sheet, while its in-plane dimensions are much larger than sheet thickness. Representative dimensions of such a sheet may be ~5 mm × 100 mm × 100 mm. We further assume that the material is nearly incompressible. Along the thickness direction, two counterpropagating acoustic waves *p*(**x**, *t*) = *p*_0_*e*^−*j*(**k**⋅**x**−*ωt*)^ (**k** is wavenumber vector, **x** position vector, and *ω* angular frequency) strike the thin sheet, governed by momentum equation ∇⋅**σ** = *ρ*∂^2^**u**/∂*t*^2^ in Eulerian coordinates, **σ** being Cauchy stress tensor. The propagation of acoustic waves with wavelength Λ = 2*πc*_*a*_/*ω* in the sheet induces acoustic radiation forces, causing it to deform from reference state (*L*_1_, *L*_2_, *L*_3_) of Fig. 1(a) to current state (*l*_1_, *l*_2_, *l*_3_) of Fig. 1(b).

For ultrasonic wave propagation in soft materials, the Cauchy stress can be obtained by incorporating the elastic deformation stress and acoustic radiation stress as

2 $${\sigma }_{ij}=\frac{{F}_{iK}}{{\rm{\det }}({\bf{F}})}\frac{\partial W({\bf{F}})}{\partial {F}_{jK}}-[\frac{\langle {p}^{2}\rangle }{2{\rho }_{a}{c}_{a}^{2}}-\frac{{\rho }_{a}\langle {u}_{k}\cdot {u}_{k}\rangle }{2}]\,{\delta }_{ij}-{\rho }_{a}\langle {u}_{i}\cdot {u}_{j}\rangle $$

where *W*(**F**) is the Helmholtz free energy corresponding to nonlinear elastic deformation of material, which is a symmetric function of deformation gradient **F**(≡∂**x**/∂**X**). If we consider the Cauchy stress as external mechanical stress, this equation actually gives the force balance condition among the external mechanical Cauchy stress, elastic deformation Cauchy stress and acoustic radiation Cauchy stress. Although it looks no explicit coupling term between the elastic deformation stress and acoustic radiation stress in Eq. (2), actually the acoustic radiation stress is highly coupled with material deformation via acoustic wave propagation in deformed configuration (see more details in Supplementary Material), which thus constitutes a nonlinear acoustomechanical coupling system. The Gent model^25^ for Helmholtz free energy of nearly incompressible materials is adopted, as:

3 $$W({\bf{F}})=-\,\frac{\mu {J}_{m}}{2}\,\mathrm{ln}(1-\frac{{I}_{1}-3}{{J}_{m}})-\mu \,\mathrm{ln}\,J+\frac{K}{2}{(J-1)}^{2}$$

where *μ* and *K* are initial shear and bulk moduli of material, *J*_*m*_ is extension limit, *I*_1_ = tr(**F**^**T**^**F**), and *J* = det(**F**). When *J*_*m*_ → ∞, the Gent model becomes the classical neo-Hookean model. Under symmetric acoustic fields (Fig. 1), force balance requires vanishing Cauchy stresses in *x*- and *y*-directions: $\frac{1}{{l}_{3}}{\int }_{0}^{{l}_{3}}{\sigma }_{1}dz=\frac{1}{{l}_{3}}{\int }_{0}^{{l}_{3}}{\sigma }_{2}dz=0$. While, the Cauchy stress in *z*-direction should balance the outside acoustic radiation as ${\sigma }_{3}=-\,\langle {T}_{33}^{outside}\rangle$. Considering these force boundary conditions, we obtain from Eq. (3) acoustic radiation induced equivalent stress, as:

4 $${\bf{t}}=\frac{2}{J}\frac{\partial W}{\partial {I}_{1}}{\bf{B}}{\boldsymbol{+}}\frac{\partial W}{\partial J}{\bf{I}}=\frac{\mu {J}_{m}}{J({J}_{m}-{I}_{1}+3)}{\bf{B}}{\boldsymbol{+}}[K(J-1)-\frac{\mu }{J}]{\bf{I}}$$

where **I** is identity tensor and **B** = **FF**^**T**^ is left Cauchy-Green deformation tensor. With reference to Fig. 1(c), the principal components of equivalent stress tensor **t** are related to the corresponding acoustic radiation stresses, as:

5 $${t}_{1}=\frac{1}{{l}_{3}}{\int }_{0}^{{l}_{3}}\langle {T}_{11}(z)\rangle dz,\,{t}_{2}=\frac{1}{{l}_{3}}{\int }_{0}^{{l}_{3}}\langle {T}_{22}(z)\rangle dz,\,{t}_{3}=\langle {T}_{33}^{inside}({l}_{3})\rangle -\langle {T}_{33}^{outside}({l}_{3})\rangle $$

We further constrain the soft material sheet to be undeformed in the *x*-direction (*i.e*., *λ*_1_ = *l*_1_/*L*_1_ = 1) and exert a mechanical force *f* in the *y*-direction, as shown schematically in Fig. 1(b). We then demonstrate that the mechanical response of soft material can be modulated by altering acoustic wave input with *f* fixed or altering *f* with acoustic wave input fixed.

## Results and Discussion

**Figure 2:**
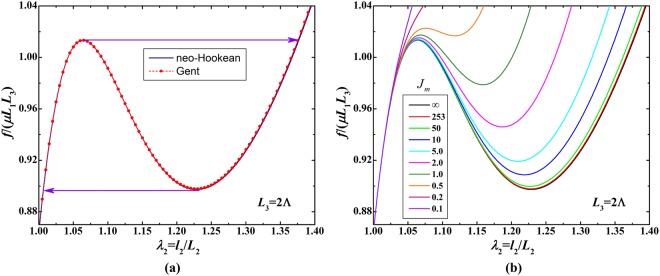
Acoustomechanical snap-through instability of soft material in response to mechanical force *f* at prescribed acoustic input of ${p}_{0}^{2}/(\mu {\rho }_{0}{c}_{0}^{2})=1.4$: (**a**) acoustomechanical hysteretic loop; (**b**) acoustomechanical responses for different extension limits *J*_*m*_.

The acoustomechanical theoretical model of compressible soft material is developed in the above section. As a large number of soft materials are nearly incompressible, the above theoretical model can be applied to incompressible soft material if the incompressible condition of *J* = det(**F**) = 1 is fully enforced. For an incompressible soft material, the relationship between normalized mechanical force *f*_*n*_ ≡ *f*/(*μL*_1_*L*_3_) and principal stretches (*λ*_1_, *λ*_2_, *λ*_3_) can be obtained by applying the constitutive relation of Eq. (4) and the incompressible condition, as:

6 $$\frac{f}{\mu {L}_{1}{L}_{3}}=[-\frac{{t}_{2}-{t}_{3}}{\mu }+\frac{({\lambda }_{2}^{2}-{\lambda }_{3}^{2})}{1-(1+{\lambda }_{2}^{2}+{\lambda }_{3}^{2}-3)/{J}_{\mathrm{lim}}}]{\lambda }_{3}$$

where *λ*_2_ = *l*_2_/*L*_2_ and *λ*_3_ = *l*_3_/*L*_3_, with ${\lambda }_{3}={\lambda }_{2}^{-1}$. For fixed acoustic input of ${p}_{0}^{2}/(\mu {\rho }_{0}{c}_{0}^{2})=1.4$, Fig. 2(a) plots *f*_*n*_ as a function of *λ*_2_ for soft material sheet with initial thickness *L*_3_ = 2Λ. The neo-Hookean model can well reproduce the Gent model in the considered stretch range. In response to variation of mechanical force at prescribed acoustic field, snap-through instability occurs in the soft material. This instability causes the stretch *λ*_2_ to jump discontinuously from a small value to a much larger one, accompanied by excessive thinning down of soft material sheet. During snapping, the normalized mechanical force first goes up, then down and then up again, showing a non-monotonic behavior. The region with negative slope in the force-stretch curve signifies negative incremental stiffness, which is unstable. If the mechanical force is uniformly distributed and controlled, a hysteresis loop would be developed in the material, as marked by the two arrows in Fig. 2(a). In practice, the hysteresis may operate in a loop smaller than (${f}_{n}^{peak}$, ${f}_{n}^{dip}$) because material defects may decrease the threshold for converting from one state to another state in a local region. Figure 2(b) presents the acoustomechanical responses of the soft material sheet with different extension limits *J*_*m*_, among which the case of *J*_*m*_ = ∞ corresponds to the neo-Hookean curve in Fig. 2(a) and the case of *J*_*m*_ = 253 corresponds to the Gent curve in Fig. 2(a). As observed in Fig. 2(b), with the decrease of the extension limit, the acoustomechanical snap-through instability tends to be suppressed. This is because the extension limit signifies the finite extensibility of polymer chains or the collective straightening of collagen fibers in biological tissues. The smaller the extension limit, the stiffener the soft material. The dramatically increased force-stretch curve of the stiffened soft material is capable of suppressing any possible instability.

**Figure 3:**
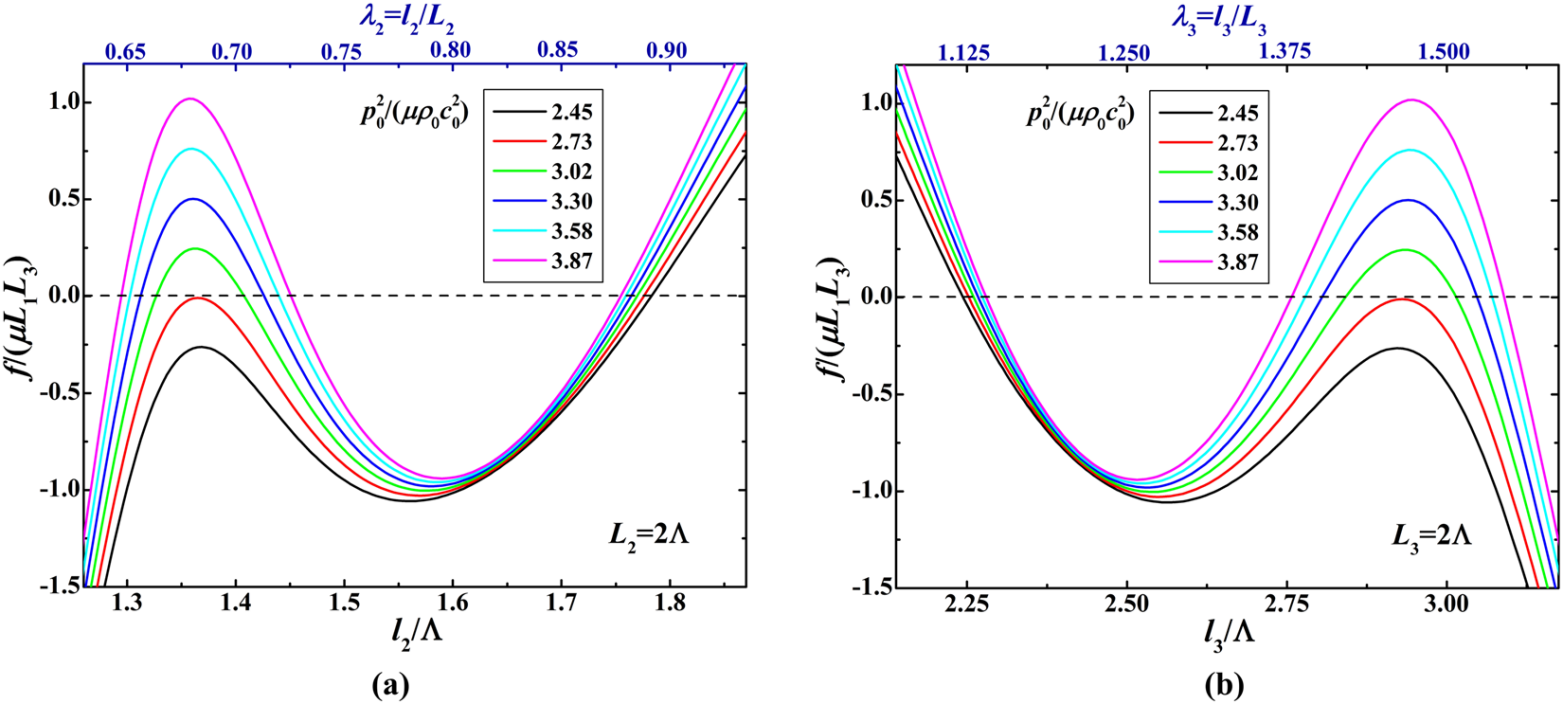
Bifurcation diagrams for the relationship between normalized mechanical force *f*/(*μL*_1_*L*_3_) and deformed dimensions triggered from different initial states: (**a**) mechanical force as a function of in-plane deformed dimension; (**b**) mechanical force as a function of out-of-plane deformed dimension.

We demonstrate that the snap-through instability of soft material in response to mechanical force can be modulated by adjusting acoustic wave inputs. Figure 3 plots the bifurcation diagrams of mechanical force as a function of deformed dimensions when triggered from different initial states. The force versus stretch curves intersect the horizontal line of zero force at different stretch points, implying the soft material is loaded at different initial states with different acoustic inputs. In more detail, as shown in Fig. 3, the different force-stretch curves correspond to different acoustic inputs ${p}_{0}^{2}/(\mu {\rho }_{0}{c}_{0}^{2})$ = (2.45, 2.73, 3.02, 3.30, 3.58, 3.87), in other words, the different acoustic inputs can programme different force-stretch relation of the soft material. Each force-stretch curve has multiple intersection points with the zero force line, these intersection points can be regarded as initial states of the mechanical force loading process. At these points, the soft material can be hold at these initial states with a steady deformation because of the acoustic radiation force generated by the fixed acoustic input. The multiple intersection points manifest the multiple steady states of the acoustomechanical response of soft material for the same acoustic input. This is because for different initial states (i.e., different current configurations), the ultrasonic wave propagation can generate different acoustic fields and the corresponding different acoustic radiation stresses, these different acoustic radiation stresses are just in balance with the elastic deformation stresses in these current configurations. It is also found from Fig. 3 that the different initial states lead to significant variation of these curves. In other words, the mechanical response of soft material can be significantly modulated by varying the acoustic input. Especially, the snap-through instability is enlarged by increasingly varying the acoustic input.

**Figure 4:**
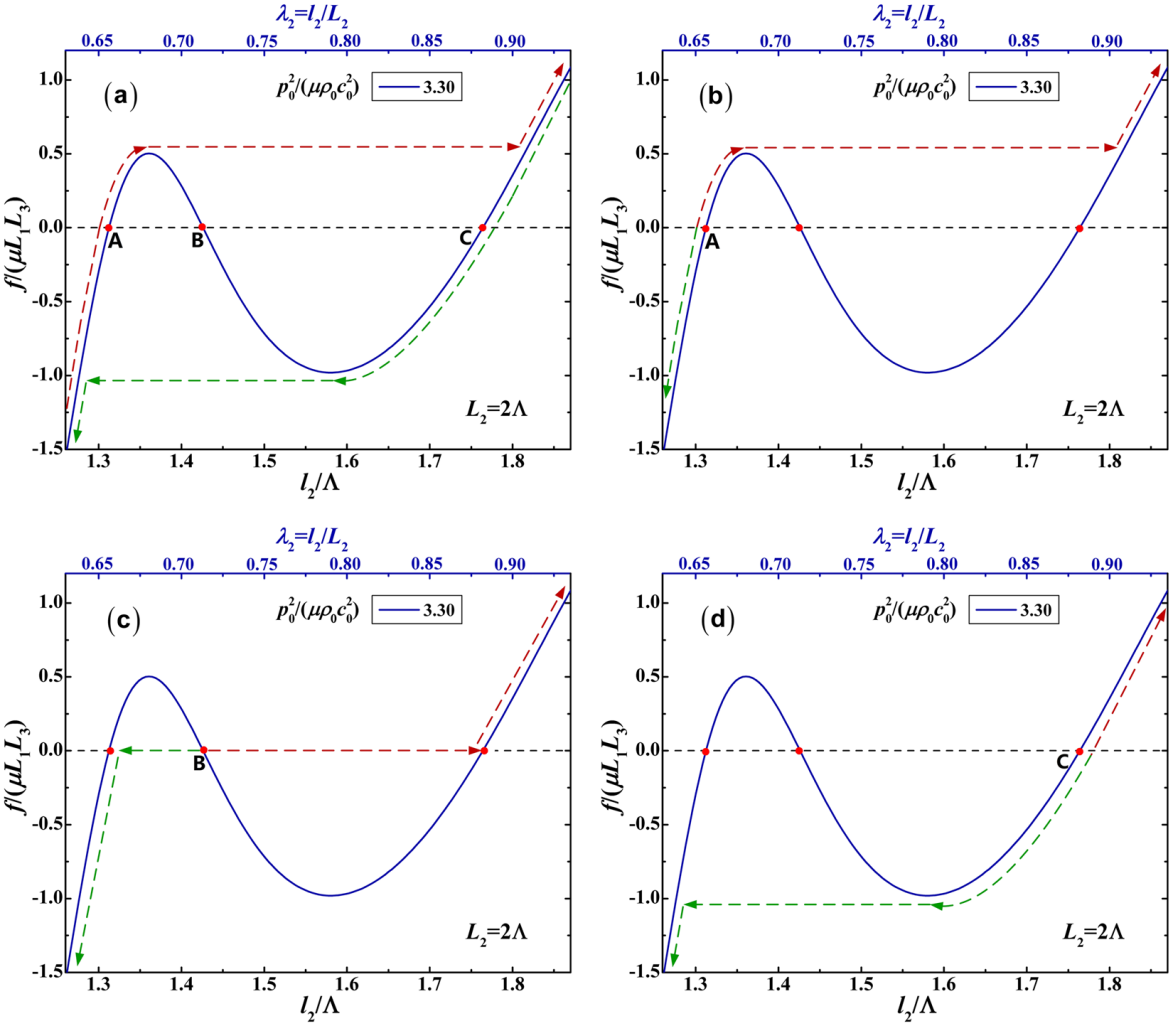
Mechanical response of soft material to mechanical force *f*/(*μL*_1_*L*_3_) as a function of in-plane deformed dimension *l*_2_/Λ (or *λ*_2_) at the specified acoustic input ${p}_{0}^{2}/(\mu {\rho }_{0}{c}_{0}^{2})=3.30$. The four responses are triggered from different initial states: (**a**) completed response; (**b**) initial state A; (**b**) initial state B; (**d**) initial state C. The red dash lines denote the positive loading path, while the green dash lines signify the unloading path (or negative loading path).

**Figure 5:**
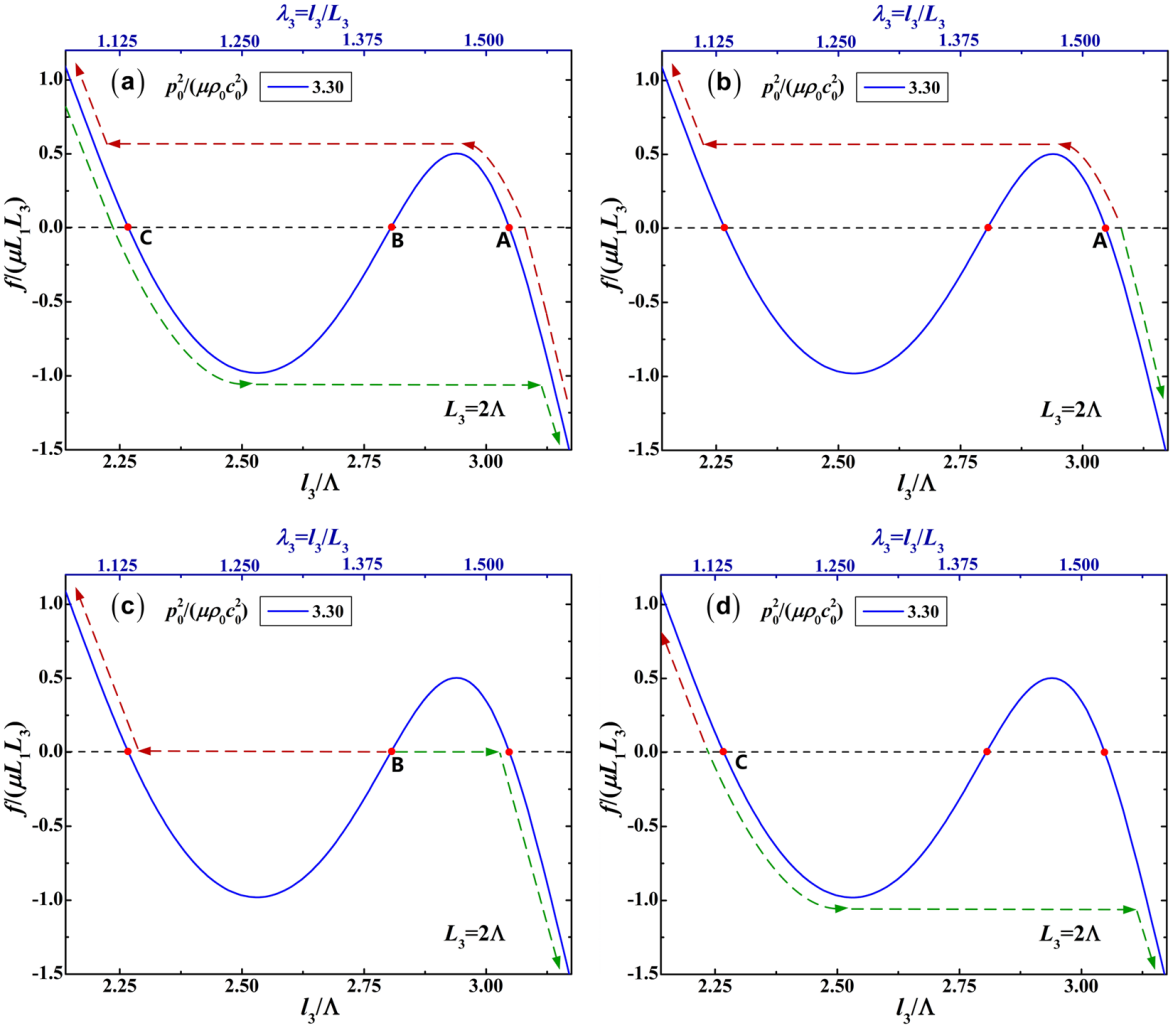
Mechanical response of soft material to mechanical force *f*/(*μL*_1_*L*_3_) as a function of out-plane deformed dimension *l*_3_/Λ (or *λ*_3_) at the specified acoustic input ${p}_{0}^{2}/(\mu {\rho }_{0}{c}_{0}^{2})=3.30$. The four responses are triggered from different initial states: (**a**) completed response; (**b**) initial state A; (**b**) initial state B; (**d**) initial state C. The red dash lines denote the positive loading path, while the green dash lines signify the unloading path (or negative loading path).

To be more specific, the mechanical response of the soft material to the mechanical force *f*/(*μL*_1_*L*_3_) at the specified acoustic input ${p}_{0}^{2}/(\mu {\rho }_{0}{c}_{0}^{2})=3.30$ is solely selected to plot as a function of the in-plane deformed dimension *l*_2_/Λ (or *λ*_2_) and the out-plane deformed dimension *l*_3_/Λ (or *λ*_3_), respectively in Figs 4 and 5. As shown in Fig. 4(a), the force-stretch curve has three intersection points A, B and C with the zero force line. Without external mechanical force, the soft material is able to hold at the deformed sates A, B and C, owing to the acoustic radiation force generated by the acoustic input ${p}_{0}^{2}/(\mu {\rho }_{0}{c}_{0}^{2})=3.30$. At these intersection points, there is no mechanical force, so the corresponding states can be considered as the initial sates of the mechanical force loading process. As shown in Fig. 4, for the in-plane deformation, the completed response, the response from initial state A, B and C are separately plotted. Correspondingly, as shown in Fig. 5, for the out-plane deformation, the completed response, the response from initial state A, B and C are separately plotted as well. In these figures, the red dash lines denote the positive loading path, while the green dash lines signify the unloading path (or negative loading path). Whatever the loading path or the unloading path, the snap-through instability can occur. From the initial state A, the force-stretch curve first goes up and then undergoes the snap-through instability in the positive loading process, or goes down in the negative loading process. From the initial state B, the force-stretch curve first undergoes the snap-through instability, and then goes up in the positive loading process, or goes down in the negative loading process. From the initial sate C, the force-stretch curve goes up in the positive loading process, or first goes down and then undergoes the snap-through instability in the negative loading process. All the force-stretch curves in Fig. 5 have the same trends as their counterpart curve in Fig. 4.

**Figure 6:**
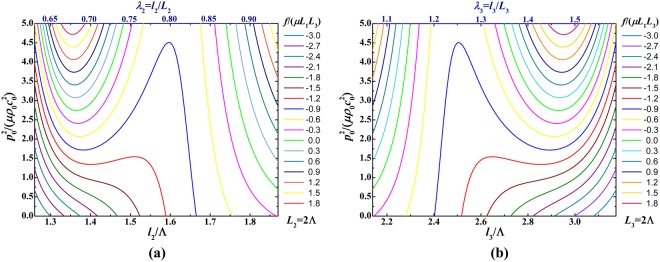
Bifurcation diagrams for relationship between acoustic input and deformed dimensions under different mechanical forces: (**a**) acoustic input as a function of in-plane deformed dimension *l*_2_/Λ (or *λ*_2_); (**b**) acoustic input as a function of out-of-plane deformed dimension *l*_3_/Λ (or *λ*_3_)

As shown in Fig. 6, analogous to acoustic modulation, bifurcation diagrams for the relationship between acoustic input and deformed dimensions under different mechanical forces indicate that the acoustical response of soft material can also be modulated by manipulating the pre-mechanical force. In these diagrams, each curve represents the mechanical response of soft material to the acoustic input at a fixed pre-mechanical force. In more detail, as shown in Fig. 6(a,b), the different acoustic input-stretch curves correspond to different pre-mechanical force, which demonstrates that the pre-mechanical force can programme the acoustic input-stretch relation of soft material. Or in other words, the acoustic input-stretch relation can be modulated by varying the pre-mechanical force. Also, it is observed from Fig. 6 that each acoustic input-stretch curve has different intersection point with the zero acoustic input line (i.e., the *x*-axis), these intersection points can be regarded as the initial state of each acoustical loading process. Without acoustical loading, the soft material can hold at a steady deformed state due to the stretch of the pre-mechanical force. From these initial states with different fixed pre-mechanical forces, each acoustic input-stretch curve tracks different acoustical loading path with the increase of the acoustic input. If we plot a horizontal line for any given acoustic input, this line will have multiple intersection points with the acoustic input-stretch curve with a fixed pre-mechanical force. These multiple intersection points manifest that there exist multiple steady states with different deformed configuration for a fixed pre-mechanical force. This is because for different deformed configurations, the ultrasonic wave propagation can generate different acoustic fields and the corresponding different acoustic radiation stresses, these different acoustic radiation stresses accompanying with the fixed mechanical stress/force are just in balance with the elastic deformation stresses in these multiple deformed configurations. Furthermore, it is found from Fig. 6 that the different pre-mechanical forces can lead to significant different acoustic input-stretch curves. In particular, the snap-through instability is remarkably enlarged as the acoustic input increases.

**Figure 7:**
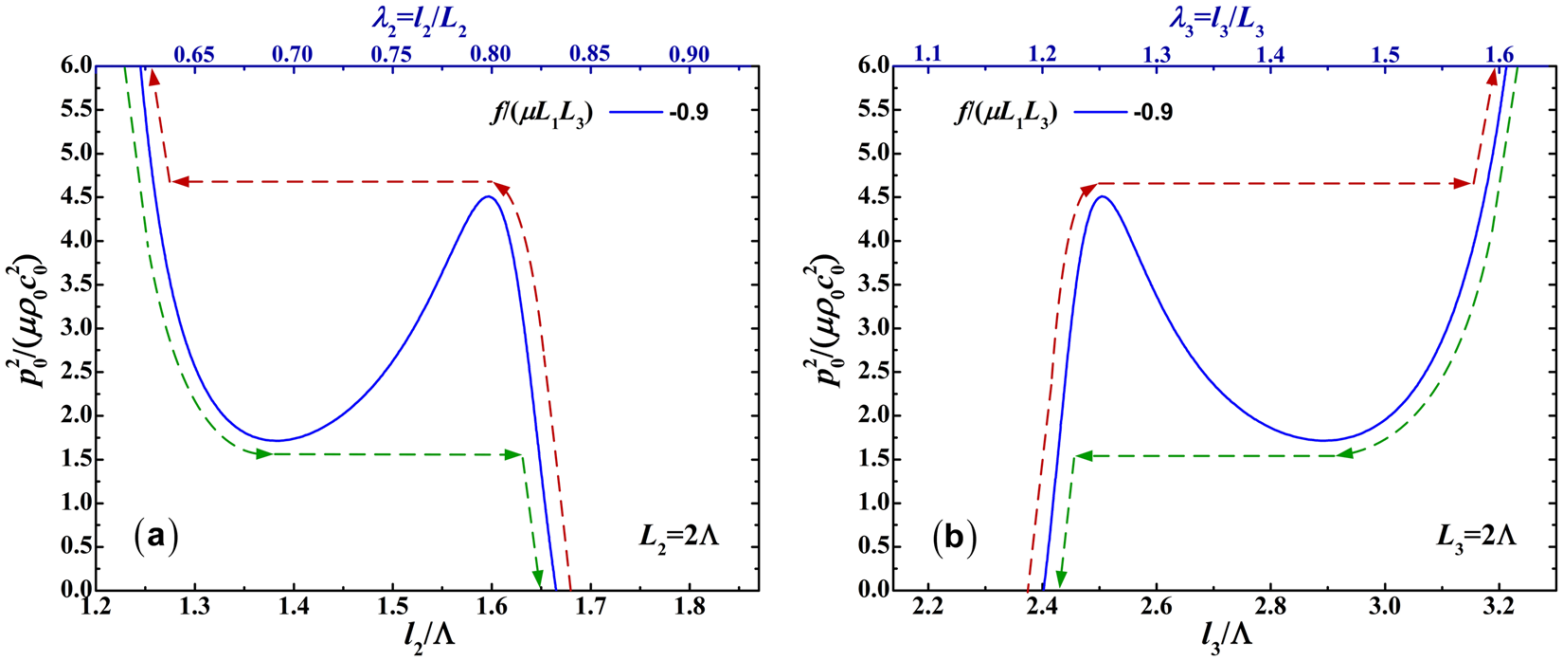
Mechanical response of soft material to acoustic input ${p}_{0}^{2}/(\mu {\rho }_{0}{c}_{0}^{2})$ at the specified mechanical force *f*/(*μL*_1_*L*_3_) = −0.9: (**a**) acoustic input as a function of in-plane deformed dimension *l*_2_/Λ (or *λ*_2_); (**b**) acoustic input as a function of out-of-plane deformed dimension *l*_3_/Λ (or *λ*_3_). The red dash lines denote the loading path, while the green dash lines signify the unloading path.

To be more specific, the acoustical response of the soft material to the acoustic input ${p}_{0}^{2}/(\mu {\rho }_{0}{c}_{0}^{2})$ at the specified mechanical force *f*/(*μL*_1_*L*_3_) = −0.9 is solely selected to plot as a function of the in-plane deformed dimension *l*_2_/Λ (or *λ*_2_) and the out-plane deformed dimension *l*_3_/Λ (or *λ*_3_), respectively in Fig. 7(a,b). In these figures, the red dash lines denote the acoustical loading path, while the green dash lines signify the unloading path. As shown in Fig. 7(a,b), the acoustic input-stretch curve has an intersection point with the zero acoustic input line, which can be considered as the initial state of the acoustic loading process. At the intersection point, without acoustic input, the soft material can hold at a steady deformed state because of the stretch of the pre-mechanical force. From the initial state, the acoustic input-stretch curve first goes up, undergoes the snap-through instability and continues to go up in the loading process. Or in the unloading process, the acoustic input-stretch curve goes down, undergoes the snap-through instability and continues to go down.

**Figure 8:**
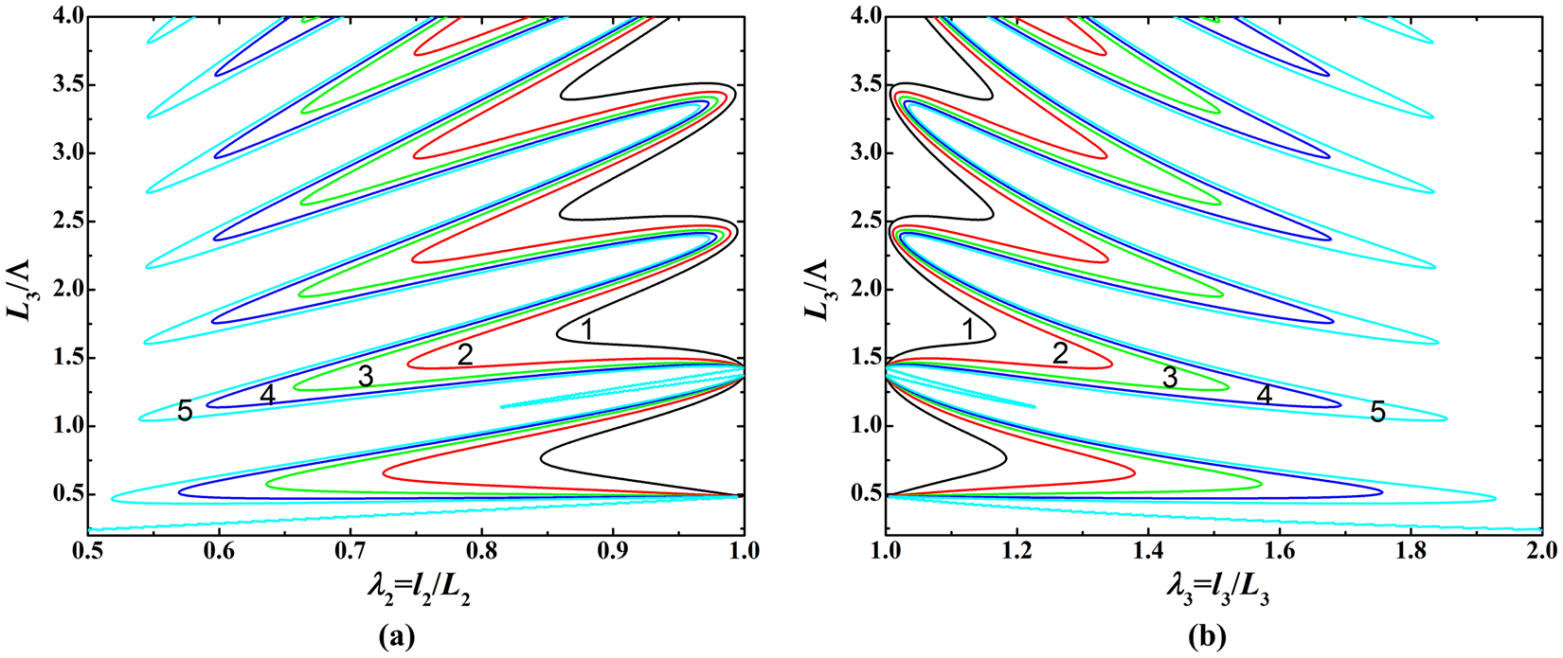
Bifurcation diagrams for relationship between initial thickness and deformed stretches of soft material sheet under fixed mechanical force *f*/(*μL*_1_*L*_3_) = 0.02 and varying acoustic loads: (**a**) initial thickness as a function of in-plane deformed stretch; (**b**) initial thickness as a function of out-of-plane deformed stretch.

Harnessing the sensitivity of acoustic wave propagation to normalized initial sheet thickness *L*_3_/Λ, we further reveal the modulated bifurcation behavior of soft material in mechanical response by exerting different acoustic inputs (Fig. 8). The ratio *L*_3_/Λ can be treated as a sole loading parameter, which exhibts load-stretch responses when different acoustic inputs are prescribed. With the initial thickness *L*_3_ fixed, this loading parameter can be readily realized by modulating the frequency (or wavelength) of inputting acoustic waves. As shown in Fig. 8, each bifurcation diagram presents five loading-stretch branches, which are associated with five prescribed acoustic inputs of ${p}_{0}^{2}/(\mu {\rho }_{0}{c}_{0}^{2})=1,\,2,\,3,\,4,\,5$. These bifurcation branches demonstrate that the load-stretch response of soft material can be modulated by altering the acoustic waves.

**Figure 9:**
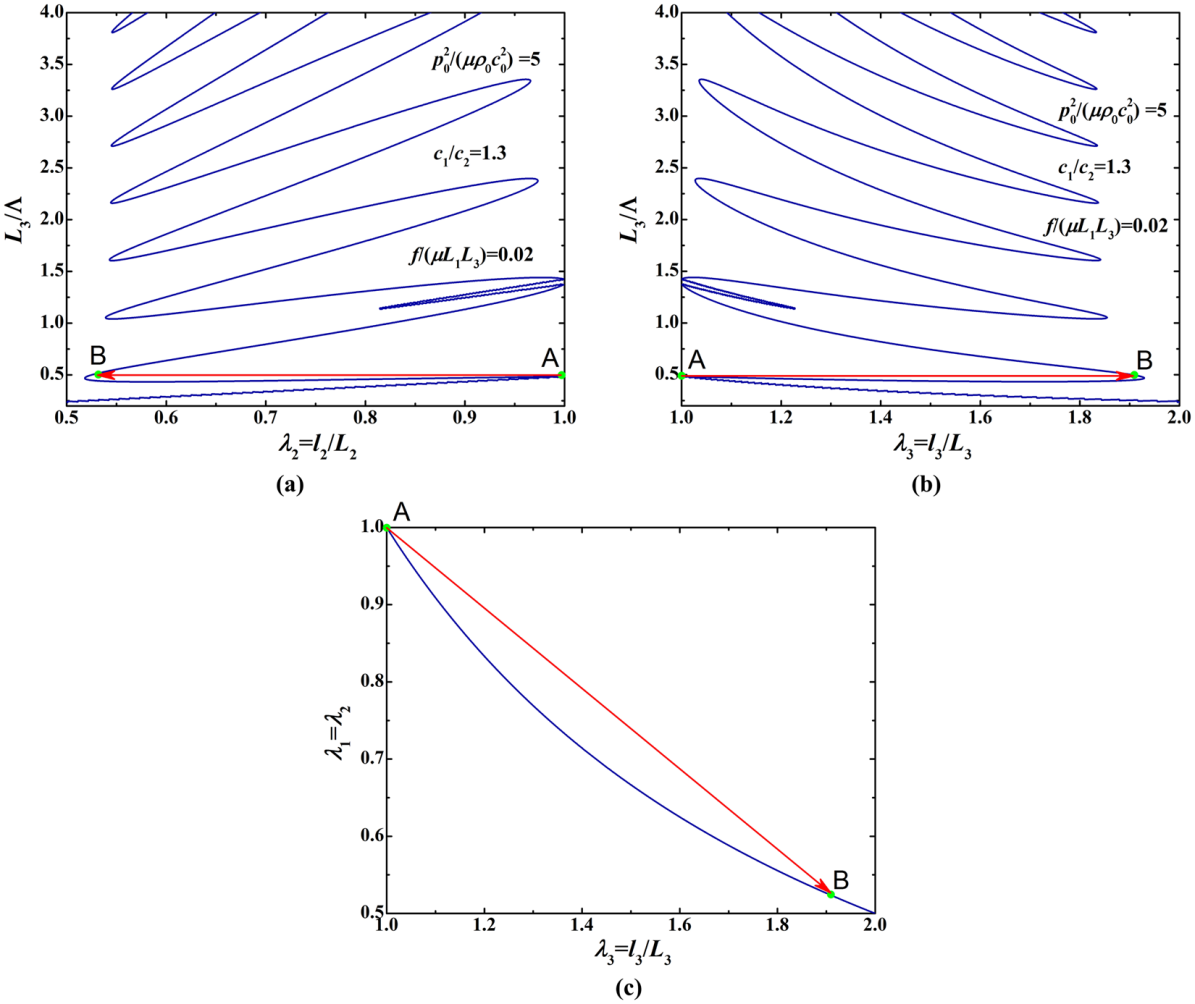
Bifurcation diagrams for relationship between initial thickness and deformed stretches of soft material sheet under fixed mechanical force *f*/(*μL*_1_*L*_3_) = 0.02 and fixed acoustic field ${p}_{0}^{2}/(\mu {\rho }_{0}{c}_{0}^{2})=5$: (**a**) initial thickness as a function of in-plane deformed stretch; (**b**) initial thickness as a function of out-of-plane deformed stretch; (**c**) in-plane stretch as a function of out-of-plane stretch.

The branch of ${p}_{0}^{2}/(\mu {\rho }_{0}{c}_{0}^{2})=5$ in Fig. 8 is individually plotted in Fig. 9 to reveal the fact that snap-through instability can also occur in bifurcation regime under the combined effect of mechanical and acoustical loading. Upon gradually increasing the loading parameter *L*_3_/Λ, the dimension of soft material jumps discontinuously from point A to point B, accompanied by a sudden and large alteration of sheet stretching, which signifies the occurrence of snap-through instability.

**Figure 10:**
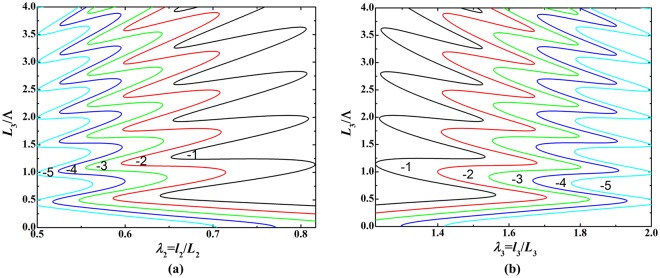
Bifurcation diagrams for relationship between initial thickness and deformed stretches under fixed acoustic field ${p}_{0}^{2}/(\mu {\rho }_{0}{c}_{0}^{2})=2$ and varying mechanical forces: (**a**) initial thickness as a function of in-plane deformed stretch; (**b**) initial thickness as a function of out-of-plane deformed stretch.

**Figure 11:**
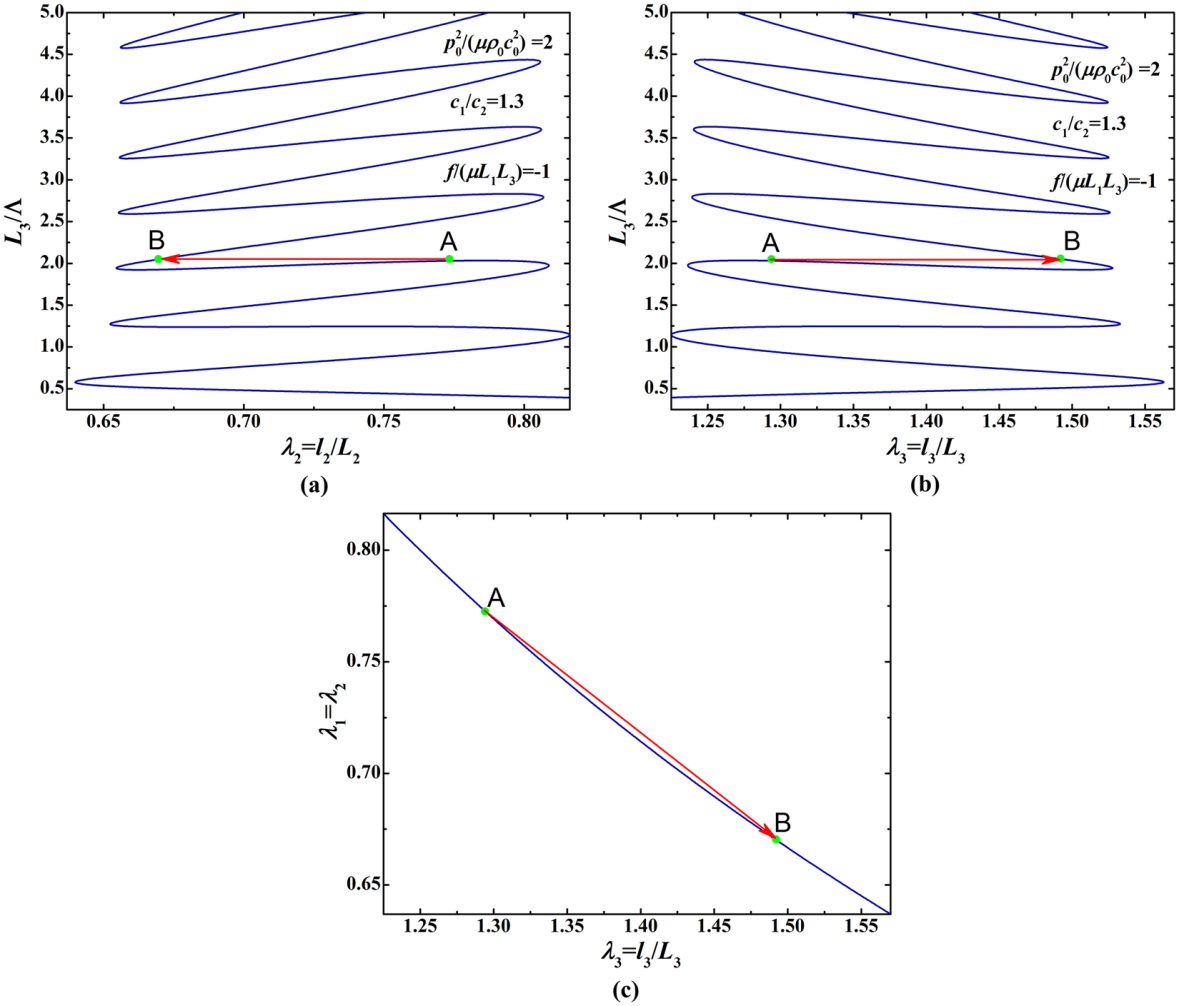
Bifurcation diagrams for relationship between initial thickness and deformed stretches under fixed acoustic field ${p}_{0}^{2}/(\mu {\rho }_{0}{c}_{0}^{2})=2$ and fixed mechanical force *f*/(*μL*_1_*L*_3_) = −1: (**a**) initial thickness as a function of in-plane deformed stretch; (**b**) initial thickness as a function of out-of-plane deformed stretch; (**c**) in-plane stretch as a function of out-of-plane stretch.

Similar to acoustic modulation, we demonstrate that the bifurcation behavior of soft material can be modulated by varying the mechanical force when prescribing the acoustic input. Figure 10 presents bifurcation diagrams for the relationship between initial thickness and deformed stretches, in which *L*_3_/Λ is treated as the sole loading parameter and each curve in these diagrams represents the load-stretch response or loading history. Each diagram of Fig. 10 show five distinct bifurcation branches related to five prescribed mechanical forces *f*/(*μL*_1_*L*_3_) = −1, −2, −3, −4, −5, implying mechanical force modulated load-stretch response. In Fig. 11, the branch of *f*/(*μL*_1_*L*_3_) = −1 is individually plotted to reveal the appearance of snap-through instability when *L*_3_/Λ is gradually increased. The deformation state discontinuously jumps from point A to point B not only in the (*L*_3_/Λ, *λ*_2_/*λ*_3_) parameter space but also in the (*λ*_2_, *λ*_3_) stretch space. This snap-through jumping realizes the direct switch from one equilibrium sate to another equilibrium state, striding over the unstable state between the two.

## Conclusions

In summary, we demonstrate the snap-through instability and bifurcation behavior of homogeneous, isotropic soft material sheets in response to not only mechanical load but also acoustic load. Snap-through instability can even show up in bifurcation branches when the ratio of initial sheet thickness to wavelength is taken as a sole loading parameter. We further uncover the feasibility of convenient modulation of acoustomechanical instability and bifurcation by altering either the mechanical or acoustic load. This new functionality of soft material enables pre-programmable response of soft material by varying the pre-mechanical force and instant-programmable response via adjusting the frequency and amplitude of acoustic input. This work would inspire innovative designs of soft actuators, microfluidic devices, adaptive robots, etc. by harnessing the tunable and programmable acoustomechanical behavior of soft materials.

## Electronic supplementary material

Supplementary material
