# Supplementary material for: Modulation of acoustomechanical instability and bifurcation behavior of soft materials
Source: Sci Rep. 2018 Nov 9;8:16661. doi: 10.1038/s41598-018-34971-x (PMC6226430; doi:10.1038/s41598-018-34971-x)
Supplement: Supplementary file 1 — Supplementary material [file 41598_2018_34971_MOESM1_ESM.pdf]

Supplementary material for "Modulation of acoustomechanical instability and bifurcation behavior of soft materials"

Fengxian Xin[1,2,*] and Tian Jian Lu[3,1,2]

[1]*State Key Laboratory for Strength and Vibration of Mechanical Structures, Xi'an Jiaotong University, Xi'an 710049, P.R. China*

[2]*MOE Key Laboratory for Multifunctional Materials and Structures, Xi'an Jiaotong University, Xi'an 710049, P.R. China*

[3]*State Key Laboratory of Mechanics and Control of Mechanical Structures, Nanjing University of Aeronautics and Astronautics, Nanjing 210016, P.R. China*

Calculation of acoustic fields and acoustic radiation stress/force

As shown in Fig. 1 of the main manuscript, consider a thin sheet of soft material immersed in a surrounding medium. A time-harmonic acoustic wave $p(\mathbf{x},t)=p_0 e^{-j(\mathbf{k}\cdot\mathbf{x}-\omega t)}$ with wavenumber vector $\mathbf{k}$ is incident on the surface of the sheet from the outside surrounding medium. Due to discontinuity of acoustic impedance at the interfaces, the incident acoustic wave will generate reflection and transmission not only on the inlet surface but also on the outlet surface. In Eulerian coordinates, wave propagation both in the material and the surrounding medium is governed by the momentum equation $\nabla\cdot\boldsymbol{\sigma}=\rho\partial^2\mathbf{u}/\partial t^2$. For ideal fluid-like materials, this equation degrades to the acoustic Helmholtz equation $\rho\partial^2\mathbf{u}/\partial t^2+\nabla p=0$. In such cases, the velocity potentials induced by the incident acoustic wave can be written as:

$$\psi_1(\mathbf{x},t)=\alpha e^{-j\left(\mathbf{k}_1^+\cdot\mathbf{x}-\omega t\right)}+\beta e^{-j\left(\mathbf{k}_1^-\cdot\mathbf{x}-\omega t\right)} \tag{1}$$

$$\psi_2(\mathbf{x},t)=\chi e^{-j\left(\mathbf{k}_2^+\cdot\mathbf{x}-\omega t\right)}+\gamma e^{-j\left(\mathbf{k}_2^-\cdot\mathbf{x}-\omega t\right)} \tag{2}$$

$$\psi_3(\mathbf{x},t)=\vartheta e^{-j\left(\mathbf{k}_3^+\cdot\mathbf{x}-\omega t\right)} \tag{3}$$

where the subscripts 1, 2 and 3 denote the left side medium, the soft material sheet, and the right side medium, while the superscripts + and – are related to positive- and negative-going waves, respectively. The corresponding velocity and pressure fields can be expressed as:

*fengxian.xin@gmail.com (F.X. Xin)

$$\mathbf{u}_i = -\nabla \psi_i ,\; p_i = \rho_i \frac{\partial \psi_i}{\partial t} \; (i=1,2,3) \tag{4}$$

Continuity of velocity and acoustic pressure at the interfaces dictates:

$$u_{1z} = u_{2z}\big|_{z=0} ,\; u_{2z} = u_{3z}\big|_{z=l_3} \tag{5}$$

$$p_1 = p_2\big|_{z=0} ,\; p_2 = p_3\big|_{z=l_3} \tag{6}$$

which can be rewritten in the form:

$$jk_{1z}\alpha e^{-j\left(k_{1x}x+k_{1y}y-\omega t\right)} - jk_{1z}\beta e^{-j\left(k_{1x}x+k_{1y}y-\omega t\right)} = jk_{2z}\chi e^{-j\left(k_{2x}x+k_{2y}y-\omega t\right)} - jk_{2z}\gamma e^{-j\left(k_{2x}x+k_{2y}y-\omega t\right)} \tag{7}$$

$$jk_{2z}\chi e^{-j\left(k_{2x}x+k_{2y}y+k_{2z}l_3-\omega t\right)} - jk_{2z}\gamma e^{-j\left(k_{2x}x+k_{2y}y-k_{2z}l_3-\omega t\right)} = jk_{3z}\vartheta e^{-j\left(k_{3x}x+k_{3y}y+k_{3z}l_3-\omega t\right)} \tag{8}$$

$$j\omega\rho_1\left[\alpha e^{-j\left(k_{1x}x+k_{1y}y-\omega t\right)} + \beta e^{-j\left(k_{1x}x+k_{1y}y-\omega t\right)}\right] = j\omega\rho_2\left[\chi e^{-j\left(k_{2x}x+k_{2y}y-\omega t\right)} + \gamma e^{-j\left(k_{2x}x+k_{2y}y-\omega t\right)}\right] \tag{9}$$

$$j\omega\rho_2\left[\chi e^{-j\left(k_{2x}x+k_{2y}y+k_{2z}l_3-\omega t\right)} + \gamma e^{-j\left(k_{2x}x+k_{2y}y-k_{2z}l_3-\omega t\right)}\right] = j\omega\rho_3\left[\vartheta e^{-j\left(k_{3x}x+k_{3y}y+k_{3z}l_3-\omega t\right)}\right] \tag{10}$$

For simplicity, assuming the same medium occupies the left side and the right side of the sot material sheet, *i.e.*, $\rho_1 = \rho_3$ and $k_1 = k_3$, one has:

$$\beta = \frac{\alpha e^{jk_{2z}l_3}\left(\rho_2^2 k_{1z}^2 - \rho_1^2 k_{2z}^2\right) + \alpha e^{-jk_{2z}l_3}\left(\rho_1^2 k_{2z}^2 - \rho_2^2 k_{1z}^2\right)}{e^{jk_{2z}l_3}\left(\rho_1 k_{2z} + \rho_2 k_{1z}\right)^2 - e^{-jk_{2z}l_3}\left(\rho_1 k_{2z} - \rho_2 k_{1z}\right)^2} \tag{11}$$

$$\chi = \frac{2\alpha\rho_1 k_{1z} e^{jk_{2z}l_3}\left(\rho_2 k_{1z} + \rho_1 k_{2z}\right)}{e^{jk_{2z}l_3}\left(\rho_1 k_{2z} + \rho_2 k_{1z}\right)^2 - e^{-jk_{2z}l_3}\left(\rho_1 k_{2z} - \rho_2 k_{1z}\right)^2} \tag{12}$$

$$\gamma = \frac{2\alpha\rho_1 k_{1z} e^{-jk_{2z}l_3}\left(\rho_1 k_{2z} - \rho_2 k_{1z}\right)}{e^{jk_{2z}l_3}\left(\rho_1 k_{2z} + \rho_2 k_{1z}\right)^2 - e^{-jk_{2z}l_3}\left(\rho_1 k_{2z} - \rho_2 k_{1z}\right)^2} \tag{13}$$

$$\vartheta = \frac{4\alpha\rho_1\rho_2 k_{1z} k_{2z} e^{jk_{1z}l_3}}{e^{jk_{2z}l_3}\left(\rho_1 k_{2z} + \rho_2 k_{1z}\right)^2 - e^{-jk_{2z}l_3}\left(\rho_1 k_{2z} - \rho_2 k_{1z}\right)^2} \tag{14}$$

We further assume two counter-propagating acoustic waves $p_L\left(z,t\right) = p_0 e^{-j\left(k_{1z}z-\omega t\right)}$ and $p_R\left(z,t\right) = p_0 e^{j\left(k_{1z}\left(z-l_3\right)+\omega t\right)}$ are normally incident on the soft material sheet (Fig. 1) so that the two input acoustic fields are symmetric with respect to its midplane. As a result, the system exhibits static deformation because the midplane remains stationary when subjected to two acoustic stresses of equal magnitude but opposing directions. The acoustic pressure and velocity can thus be expressed as:

$$p_1 = j\omega\rho_1\alpha e^{j\omega t}\left[e^{-jk_{1z}z} + e^{jk_{1z}z}\frac{j\sin(k_{2z}l_3)\left(\rho_2^2k_{1z}^2 - \rho_1^2k_{2z}^2\right) + 2\rho_1\rho_2k_{1z}k_{2z}}{2\rho_1\rho_2k_{1z}k_{2z}\cos(k_{2z}l_3) + j\left(\rho_1^2k_{2z}^2 + \rho_2^2k_{1z}^2\right)\sin(k_{2z}l_3)}\right] \tag{15}$$

$$u_{1z} = jk_{1z}\alpha e^{j\omega t}\left[e^{-jk_{1z}z} - e^{jk_{1z}z}\frac{j\sin(k_{2z}l_3)\left(\rho_2^2k_{1z}^2 - \rho_1^2k_{2z}^2\right) + 2\rho_1\rho_2k_{1z}k_{2z}}{2\rho_1\rho_2k_{1z}k_{2z}\cos(k_{2z}l_3) + j\left(\rho_1^2k_{2z}^2 + \rho_2^2k_{1z}^2\right)\sin(k_{2z}l_3)}\right] \tag{16}$$

$$p_2 = \frac{2\omega\alpha\rho_1\rho_2k_{1z}e^{j\omega t}\left[\rho_2k_{1z}\sin\left(k_{2z}(z-l_3)\right) - \rho_2k_{1z}\sin(k_{2z}z) + j\rho_1k_{2z}\cos\left(k_{2z}(z-l_3)\right) + j\rho_1k_{2z}\cos(k_{2z}z)\right]}{2\rho_1\rho_2k_{1z}k_{2z}\cos(k_{2z}l_3) + j\left(\rho_1^2k_{2z}^2 + \rho_2^2k_{1z}^2\right)\sin(k_{2z}l_3)} \tag{17}$$

$$u_{2z} = \frac{2\alpha\rho_1k_{1z}k_{2z}e^{j\omega t}\left[\rho_1k_{2z}\sin\left(k_{2z}(z-l_3)\right) + \rho_1k_{2z}\sin(k_{2z}z) + j\rho_2k_{1z}\cos\left(k_{2z}(z-l_3)\right) - j\rho_2k_{1z}\cos(k_{2z}z)\right]}{2\rho_1\rho_2k_{1z}k_{2z}\cos(k_{2z}l_3) + j\left(\rho_1^2k_{2z}^2 + \rho_2^2k_{1z}^2\right)\sin(k_{2z}l_3)} \tag{18}$$

$$p_3 = j\omega\rho_1\alpha e^{j\omega t}\left[e^{jk_{1z}(z-l_3)} + e^{-jk_{1z}(z-l_3)}\frac{j\sin(k_{2z}l_3)\left(\rho_2^2k_{1z}^2 - \rho_1^2k_{2z}^2\right) + 2\rho_1\rho_2k_{1z}k_{2z}}{2\rho_1\rho_2k_{1z}k_{2z}\cos(k_{2z}l_3) + j\left(\rho_1^2k_{2z}^2 + \rho_2^2k_{1z}^2\right)\sin(k_{2z}l_3)}\right] \tag{19}$$

$$u_{3z} = jk_{1z}\alpha e^{j\omega t}\left(-e^{jk_{1z}(z-l_3)} + e^{-jk_{1z}(z-l_3)}\frac{j\sin(k_{2z}l_3)\left(\rho_2^2k_{1z}^2 - \rho_1^2k_{2z}^2\right) + 2I\rho_1\rho_2k_{1z}k_{2z}}{2\rho_1\rho_2k_{1z}k_{2z}\cos(k_{2z}l_3) + j\left(\rho_1^2k_{2z}^2 + \rho_2^2k_{1z}^2\right)\sin(k_{2z}l_3)}\right) \tag{20}$$

Once the pressure and velocity are known, the acoustic radiation stress tensors both inside and outside the material can be calculated by time-averaging the corresponding variables, as:

$$\left\langle \mathbf{T}^{outside}\right\rangle \equiv \left[\frac{\left\langle p_1^2\right\rangle}{2\rho_1c_1^2} - \frac{\rho_1\left\langle \mathbf{u}_1\cdot\mathbf{u}_1\right\rangle}{2}\right]\mathbf{I} + \rho_1\left\langle \mathbf{u}_1\otimes\mathbf{u}_1\right\rangle \tag{21}$$

$$\left\langle \mathbf{T}^{inside}\right\rangle \equiv \left[\frac{\left\langle p_2^2\right\rangle}{2\rho_2c_2^2} - \frac{\rho_2\left\langle \mathbf{u}_2\cdot\mathbf{u}_2\right\rangle}{2}\right]\mathbf{I} + \rho_2\left\langle \mathbf{u}_2\otimes\mathbf{u}_2\right\rangle \tag{22}$$

where the subscripts "1, 2" denote the outside medium and the inside medium, respectively.

Applying the formulation of acoustic radiation stress, we obtain

$$\left\langle T_{11}^{outside}\right\rangle = \left\langle T_{22}^{outside}\right\rangle = \frac{A_{c1}B_{c1}^*e^{-2jk_{2z}z} + A_{c1}^*B_{c1}e^{2jk_{2z}z}}{2\rho_1c_1^2},\ \left\langle T_{33}^{outside}\right\rangle = \frac{A_{c1}A_{c1}^* + B_{c1}B_{c1}^*}{2\rho_1c_1^2} \tag{23}$$

$$\left\langle T_{11}^{inside}\right\rangle = \left\langle T_{22}^{inside}\right\rangle = \frac{A_{c2}B_{c2}^*e^{-2jk_{2z}z} + A_{c2}^*B_{c2}e^{2jk_{2z}z}}{2\rho_2c_2^2},\ \left\langle T_{33}^{inside}\right\rangle = \frac{A_{c2}A_{c2}^* + B_{c2}B_{c2}^*}{2\rho_2c_2^2} \tag{24}$$

with

$$A_{c1} = j\omega\rho_1\alpha e^{jk_{1z}l_3}\left(\frac{j\left(\rho_2^2k_{1z}^2 - \rho_1^2k_{2z}^2\right)\sin(k_{2z}l_3) + 2\rho_1\rho_2k_{1z}k_{2z}}{2\rho_1\rho_2k_{1z}k_{2z}\cos(k_{2z}l_3) + j\left(\rho_1^2k_{2z}^2 + \rho_2^2k_{1z}^2\right)\sin(k_{2z}l_3)}\right) \tag{25}$$

$$B_{c1} = j\omega\rho_1\alpha e^{-jk_{1z}l_3} \tag{26}$$

$$A_{c2} = j\omega\rho_1\rho_2 k_{1z}\alpha\left(\frac{e^{jk_{2z}l_3}\left(\rho_2 k_{1z}+\rho_1 k_{2z}\right)+\left(\rho_1 k_{2z}-\rho_2 k_{1z}\right)}{2\rho_1\rho_2 k_{1z}k_{2z}\cos\left(k_{2z}l_3\right)+j\left(\rho_1^2 k_{2z}^2+\rho_2^2 k_{1z}^2\right)\sin\left(k_{2z}l_3\right)}\right) \tag{27}$$

$$B_{c2} = j\omega\rho_1\rho_2 k_{1z}\alpha\left(\frac{e^{-jk_{2z}l_3}\left(\rho_1 k_{2z}-\rho_2 k_{1z}\right)+\left(\rho_2 k_{1z}+\rho_1 k_{2z}\right)}{2\rho_1\rho_2 k_{1z}k_{2z}\cos\left(k_{2z}l_3\right)+j\left(\rho_1^2 k_{2z}^2+\rho_2^2 k_{1z}^2\right)\sin\left(k_{2z}l_3\right)}\right) \tag{28}$$

where (T_{11}^{inside}, T_{22}^{inside}, T_{33}^{inside}) and ($T_{11}^{outside}$, $T_{22}^{outside}$, $T_{33}^{outside}$) is the acoustic radiation stress inside and outside the soft material in x-, y- and z-direction, respectively. As shown in Fig. 2(b), the equivalent stresses can be obtained by homogenizing the acoustic radiation stresses, as:

$$t_1 = \frac{1}{l_3}\int_0^{l_3}\left\langle T_{11}^{inside}\left(z\right)\right\rangle dz\,,\ t_2 = \frac{1}{l_3}\int_0^{l_3}\left\langle T_2^{inside}\left(z\right)\right\rangle dz\,,\ t_3 = \left\langle T_{33}^{inside}\left(l_3\right)\right\rangle - \left\langle T_{33}^{outside}\left(l_3\right)\right\rangle \tag{29}$$

where l_3 is the thickness of the sheet, ($\left\langle T_{11}(z)\right\rangle$, $\left\langle T_{22}(z)\right\rangle$, $\left\langle T_{33}(z)\right\rangle$) are the acoustic radiation stresses in the principal directions obtained by applying time-averaged manipulation of momentum flux over a cycle, *i.e.*, $\left\langle \mathbf{T}\right\rangle = \left(\omega/2\pi\right)\int_0^{2\pi/\omega}\mathbf{T}dt$, and ($t_1$, t_2, t_3) are the corresponding equivalent stresses, as shown in Fig. 1(b). The superscripts "inside" and "outside" denotes variables related to the inside material and the outside surrounding medium, respectively. If the acoustic fields are assumed to only occupy the in-plane region of the soft material sheet, the equivalent stresses t_1 and t_2 only contain the inside material part, while the equivalent stress t_3 contains both the inside material part and the outside surrounding medium part. Therefore, the corresponding equivalent acoustic radiation stresses are

$$t_1 = t_2 = \frac{1}{2\rho_2 c_2^2}\left\{\frac{1}{2jk_{2z}l_3}\left[A_{c2}^{*}B_{c2}\left(e^{2jk_{2z}l_3}-1\right)-A_{c2}B_{c2}^{*}\left(e^{-2jk_{2z}l_3}-1\right)\right]\right\} \tag{30}$$

$$t_3 = \frac{A_{c2}A_{c2}^{*}+B_{c2}B_{c2}^{*}}{2\rho_2 c_2^2} - \frac{A_{c1}A_{c1}^{*}+B_{c1}B_{c1}^{*}}{2\rho_1 c_1^2} \tag{31}$$

here the superscript asterisk * means the complex conjugates of the corresponding variables. These equivalent stresses are the resultant stresses that cause large deformation in the soft material.
